# Studying large-scale brain networks: electrical stimulation and neural-event-triggered fMRI

**DOI:** 10.1186/1471-2202-14-S1-A1

**Published:** 2013-07-08

**Authors:** NK Logothetis, O Eschenko, Y Murayama, M Augath, T Steudel, HC Evrard, M Besserve, A Oeltermann

**Affiliations:** 1 Max Planck Institute for Biological Cybernetics, Spemannstraße 38, 72076 Tuebingen, Germany; 2 Centre for Imaging Sciences, Biomedical Imaging Institute, The University of Manchester, Manchester M13 9PT, UK; 3 Max Planck Institute for Intelligent Systems, Spemannstraße 38, 72076 Tuebingen, Germany

## 

The brain is "the" example of an adaptive, complex system. It is characterized by ultra-high structural complexity and massive connectivity, both of which change and evolve in response to experience. Information related to sensors and effectors is processed in both a parallel and a hierarchical fashion. The connectivity between different hierarchical levels is bidirectional, and its effectiveness is continuously controlled by specific associational and neuromodulatory centers. In the study of such systems one major problem is the adequate definition for an elementary operational unit (often called an "agent"), because any such module can be a complex system in its own right and may be recursively decomposed into other sets of units. A second difficulty arises from the synergistic organization of complex systems and of the brain in particular. Synergy here refers to the fact that the behavior of an integral, aggregate, whole system cannot be trivially reduced to, or predicted from, the components themselves. Localizing and comprehending the neural mechanisms underlying our cognitive capacities demands the combination of multimodal methodologies, i.e. it demands concurrent study of components and networks; one way of doing this, is to combine invasive methods which afford us direct access to the brain's electrical activity at the microcircuit level with global imaging technologies such as magnetic resonance imaging (MRI). In my talk, I'll discuss two such methodologies: Direct Electrical Stimulation and fMRI (DES-fMRI) and Neural-Event-Triggered fMRI (NET-fMRI).

DES-fMRI can be used in hopes of gaining insight into the functional or effective connectivity underlying DES-induced behaviors. Yet, our first findings suggest that DES has an important limitation: It clearly demarcates all monosynaptic targets of a stimulated site, but it largely fails to reveal polysynaptic cortico-cortical connectivity.

NET-fMRI, on the other hand, appears to offer great potential for mapping whole-brain activity that is associated with individual local events. In the second part of my talk, I'll describe the characteristic states of widespread cortical and subcortical networks that are associated with the occurrence of hippocampal sharp waves and ripples; the brief aperiodic episodes associated with memory consolidation.

